# The impact of eccentric training on athlete movement speed: a systematic review

**DOI:** 10.3389/fphys.2024.1492023

**Published:** 2024-12-13

**Authors:** Yugang Zhang, Yuan Zhuang, Lei Zhang, Min Sun

**Affiliations:** ^1^ Department of Sports, Yuncheng University, Yuncheng, China; ^2^ Department of Physical Education College of Physical Education Yeungnam university Gyeongsan, Gyeongsan-si, Republic of Korea

**Keywords:** eccentric training, movement speed, sport-specific skills, training intervention, change of direction (COD) speed

## Abstract

Eccentric training has been proposed as a potential method for enhancing athletes' movement speed. However, a systematic review specifically examining the impact of eccentric training on movement speed in athletes has yet to be conducted. This paper aims to fill this gap by evaluating the effects of eccentric training on movement speed in athletes.A systematic search was carried out on May 15, 2024, in accordance with the PRISMA guidelines for systematic reviews and meta-analyses. The search was conducted across multiple databases, including Web of Science, EBSCOhost, PubMed, CNKI, and VIP, using keywords related to eccentric training, athletes, and movement speed. Out of 421 reviews screened, nine met the inclusion criteria. The quality of the studies was assessed using the PEDro scale, with an average score of 3.67 (range 3–4). The results indicated that short-distance sprint speed (n = 7) was the most affected by eccentric training interventions. Sport-specific movement speed (n = 3) and change of direction speed (COD) (n = 5) were also positively influenced, although to a lesser extent.In conclusion, eccentric training significantly enhances sport-specific movement speed and COD speed, with more moderate effects on short-distance sprinting. These findings suggest that eccentric training can be a valuable intervention for improving movement speed in athletes, especially for sports that involve rapid directional changes.

**Systematic Review Registration:**

https://www.crd.york.ac.uk/prospero/display_record.php?ID=CRD42024547112, identifier CRD42024547112.

## Introduction

Excellence in athletic performance relies on the mastery of proficient motor skills, strong psychological resilience, and exceptional physical fitness (Sommerfield et al., 2022). Among these factors, physical fitness is key to achieving outstanding athletic performance (Kuroda et al., 2015). Within physical fitness, an athlete’s movement speed is crucial for optimal technical performance and closely correlates with the ability to execute offensive or defensive strategies. A decrease in movement speed can affect performance and increase injury risk (Pavei and La Torre, 2016). For example, in high-intensity soccer matches, insufficient movement speed limits dribbling and defensive abilities, impacting both team performance and raising the likelihood of injuries due to exertion (Mjølsnes et al., 2004). 

Existing literature has established a correlation between training interventions and movement speed. Resistance training and strength training are frequently employed in physical education, athlete training, and research to enhance movement speed (McEvoy and Newton, 1998; Kotzamanidis et al., 2005; Bosch and Cook, 2015). However, some studies suggest that resistance training has limited impact on athletes’ movement speed (Blagrove, Howatson and Hayes, 2018; Balsalobre-Fernández, Santos-Concejero and Grivas, 2016). Researchers argue that while resistance training increases muscle strength and explosiveness, it may also hinder technical execution and impose additional muscle strain (Ross and Leveritt, 2001; Bolger et al., 2015).

Conversely, eccentric training, frequently used in medical rehabilitation (Wang et al., 2024) and tendonopathy treatment (Murtaugh and Ihm, 2013), is thought to improve posture and movement efficiency more effectively than traditional resistance training (Katsura et al., 2019), reduce injury risk (Čretnik et al., 2022), and enhance flexibility (O’Sullivan et al., 2012). Other studies also reveal that eccentric training improves movement speed in volleyball (Zhang, 2016), tennis (Zhang et al., 2019), and sprinting speed in soccer (di Cagno et al., 2020). Despite the positive impact of eccentric training on movement speed and injury reduction, there remains a lack of comprehensive information on its effects specifically on athletes’ movement speed.

Conceptually, eccentric training involves slow, controlled muscle extension under external load, ensuring continuous resistance throughout the movement. This type of training, emphasizing slow movements and load control, effectively activates muscle fibers, enhancing muscle strength and endurance (Kravitz and Bubbico, 2015). Eccentric training modalities include weighted descent, controlled stretching, loaded stretching, and eccentric contraction, making it applicable across various fields and training needs. Its essence lies in muscle contraction under load, stimulating muscle adaptation and increasing strength and endurance (Tinwala et al., 2017). Thus, eccentric training differs from traditional training in terms of muscle contraction mode and training outcomes, promoting muscle growth, strength, and endurance (Douglas et al., 2017).

As research progresses, the application range of eccentric training broadens, with scholars achieving expected outcomes by aligning training goals with eccentric training through various training protocols (Roig et al., 2009; Ansari et al., 2023). This indicates that correctly applying professional training protocols enhances the likelihood of achieving anticipated results. For example, eccentric training programs developed for rehabilitation rapidly improve health in target populations (Frizziero et al., 2014). The varying effects of eccentric training on athletes’ movement speed in existing research are promising, suggesting different training methods yield unique impacts. Therefore, this systematic review aims to clarify the influence of eccentric training on athletes’ movement speed.

## Methods

### Protocol and registration

The selection, collection, and analysis of data for this review followed the PRISMA (Preferred Reporting Items for Systematic Reviews and Meta-Analyses) guidelines and was prospectively registered on the International Prospective Register of Systematic Reviews: https://www.crd.york.ac.uk/prospero/display record.php?ID=42024547112.

### Literature search strategy

The literature search was conducted across five databases: Web of Science, EBSCOhost, PubMed, CNKI, and VIP. Each database was searched by title and abstract. The search was carried out on 3 April 2024, using keyword combinations: (eccentric overload training OR eccentric overload OR eccentric training OR eccentric load OR eccentric exercise) AND (movement speed OR speed OR athletic performance OR exercise OR mobility OR deceleration) AND (randomized controlled trial OR randomized control OR experiment OR randomized controlled study OR randomized experiment). Additional relevant articles from reference lists were also reviewed, and reference lists of previous reviews were examined to include relevant studies.

### Eligibility criteria

The literature search strategy for this review used the PICOS (Population, Intervention, Comparison, Outcomes, Study Design) criteria as inclusion standards, as shown in Table 1. A study was included if it met the following requirements.

1. Population: The study population must include professional and non-professional athletes, regardless of age, sport type, sex, sample size, or intervention duration (minimum 4 weeks), with no restriction on location.
2. Intervention: The study must include a planned and structured eccentric training intervention aimed at improving or maintaining athletes’ movement speed or sport-specific movement speed.
3. Publication: Only full-text articles published after 2000 were considered, focusing on studies describing the effects of eccentric training on athletes’ movement speed, including randomized controlled trials, non-randomized controlled trials, and single-group trials.
4. Outcome: Outcomes must include at least one measure of the effect of eccentric training on athletes’ movement speed.
5. Study Design: Studies must be experimental in nature, including single-group trials or randomized controlled trials.

**TABLE 1:** Inclusion criteria according to the PICOS conditions.

Project	Detailed inclusion criteria
Population	Athletes (male, female)
Intervention	Eccentric Training
Comparison	Experiments involving two or more groups and single-group trials
Outcome	Movement Speed
Study Design	Randomized Controlled Trials or Non-Randomized Controlled Trials

### Studies were excluded if they met the following criteria

1. Intervention: The intervention involved non-exercise training elements alongside eccentric training (e.g., pharmacological, psychological interventions).
2. Publication Type: Short-form papers such as conference proceedings, dissertations, and report papers were excluded.

### Study selection

The identified literature was imported into the Note Express reference management software for document management. Initially, the search process involved experienced professionals to aid in identifying and removing duplicate studies. Then, two authors (ZYG and ZY) independently screened titles and abstracts, assessing studies for inclusion. In cases of disagreement, a third author (ZL) was consulted to reach a consensus.

### Data extraction and quality assessment

Upon completion of the search, information was extracted from eligible studies, including: 1) basic study information (author, title, publication year); 2) sample characteristics (age, sex, quantity, sport); 3) intervention details (type, measurement indicators, duration); 4) study design; and 5) study outcomes. One author (ZY) standardized the extracted information, while another author (ZL) reviewed it for accuracy.

The PEDro scale, with established reliability and validity, was used to assess the quality of methodology and reporting in the selected studies (Sherrington et al., 2000). This tool evaluates trial quality across five domains: randomization, blinding, inter-group comparison, data handling precision, and reporting completeness (Lima et al., 2013). The PEDro scale consists of 11 items, with a scoring range of 0–10 points (8–10 indicates excellent quality; 5–7 good quality; 3–4 moderate quality; below three poor quality). Higher PEDro scores indicate better methodological quality (Foley et al., 2003). Two authors (ZYG and SM) independently scored the selected studies based on the 11 criteria using a “yes” or “no” system. Discrepancies were resolved by involving a third author (ZL).

## Results

Studies were screened and read according to the inclusion and exclusion criteria. Nine articles met the criteria, examining the effects of eccentric training on athletes’ movement speed, published between 2009 and 2022. Table 2 provides an overview of each study’s characteristics and findings.

**TABLE 2:** Characteristics of sample studies in this review.

Study	Population	Intervention						Main outcome related to skills
	N	Type of athletes	Sex	Age	Type	Skill measured index	Frequency and duration	
Zhang et al. (2019)	36	tennis players	Male	15.9 ± 0. 6 years	EG: Functional training + Eccentric training CG: Functional training	30 m Fan-shaped movement (COD) 10-m approach followed by deceleration Sport-specific movement speed	6 weeks, twice a week, 45 min each time	30 m↔ Fan-shaped movement (COD)↑ 10 m↑; approach followed by deceleration↑ Sport-specific movement speed↑
Zhang (2016)	28	Volleyball players	Female	EG:22.78 ± 1. 07 years CG:22.69 ± 0. 92 years	EG: Eccentric training CG: Traditional training	Sport-specific movement speed	8 weeks, twice a week, 130 min each time	Sport-specific movement speed↑
Jönhagen et al. (2009)	28	soccer players	Male	average 18 (17–20)years	EG: Physical training + Eccentric training CG: Regular physical training	30 m	8 weeks, four times a week	30 m↑
Fiorilli et al. (2020)	34	soccer players	Male	EG:13.21 ± 1.21 years CG:13.36 ± 0.80 years	EG: Conventional training + Eccentric training CG: Conventional training + Resistance training	Change of direction (COD) 60 m Sport-specific skills	6 weeks, twice a week, 120 min each time	Change of direction (COD)↑ 60 m↑ Sport-specific skills↑
Chaabene et al. (2022)	19	Handball player	Female	EG:20.50 ± 2.5 years CG:20.60 ± 1.6 years	EG: Conventional handball training + Eccentric training CG: Conventional handball training	5 m 10 m 20 m Change of direction (COD)	8 weeks, 5–6 times per week, approximately 8 h per week	5 m↑ 10 m↑ 20 m↑ Change of direction (COD)↑
di Cagno et al. (2020)	54	Fencers	Male	EG:17.6 ± 2.7 years CG:17.3 ± 1.9 years	EG: Eccentric training CG: Conventional training	Duration of sprinting while carrying a saber Distance of sprinting while carrying a saber Duration of sprinting without carrying a saber Distance of sprinting without carrying a saber	6 weeks, twice a week, 60 min each time	Duration of sprinting while carrying a saber↑ Distance of sprinting while carrying a saber↑ Duration of sprinting without carrying a saber↑ Distance of sprinting without carrying a saber↑
Sagelv et al. (2020)	25	soccer players	Male	EG:23.07 ± 3.15 years CG:23.07 ± 3.15 years	EG: Eccentric training CG: Conventional training	10 m	6 weeks, three times a week	10 m↑
O’Brien et al. (2020)	20	Basketball players	Female	EG:23.17 ± 5.55 CG:24.18 ± 6.56 years	EG:Eccentric training CG: Weighted squat training	10 m Change of direction (COD)	4 weeks, twice a week	10 m↑ Change of direction (COD)↑
Stojanović et al. (2021)	24	Basketball players	Male	EG:17.6 ± 0.5 years CG:17.5 ± 0.6 years	EG:Eccentric overload training CG:Traditional strength training	5 m 20 m *t*-test (COD)	8 weeks, 1–2 times per wee	5 m↑ 20 m↔ *t*-test (COD)↑

EG, Experimental Group; CG, Control Group; ↑, significant improvement; ↔, no significant difference.

### Study selection

**FIGURE 1:**
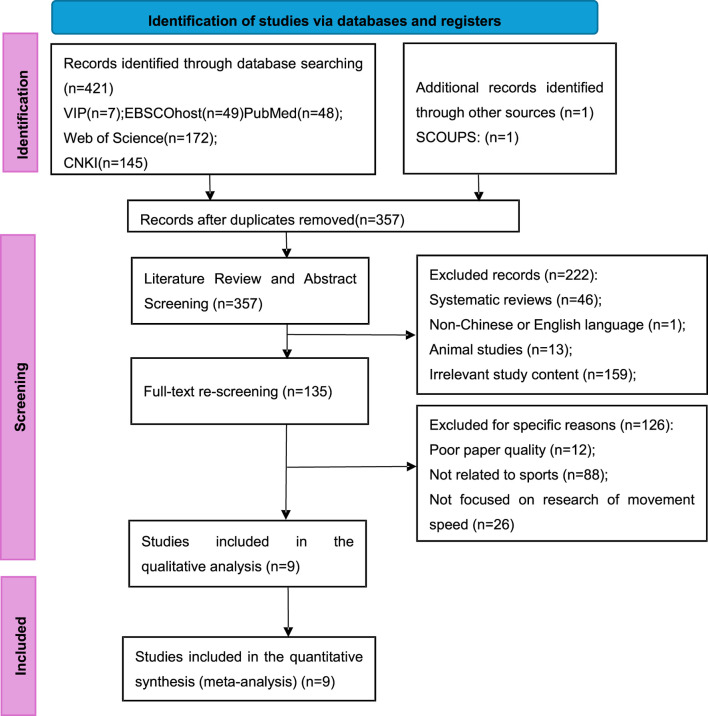
The search, screening and selection processes for suitable studies–based on PRISMA.

Figure 1 illustrates the PRISMA flow diagram of the study selection process. A total of 421 potential articles were identified through the search strategy (VIP: 7; EBSCOhost: 49; PubMed: 48; Web of Science: 172; CNKI: 145), with an additional record retrieved from other sources (SCOUPS: 1). After removing duplicates, 357 articles remained for title and abstract screening. During the title and abstract screening phase, 222 articles were excluded for the following reasons: systematic reviews (46), non-English or non-Chinese language (1), animal studies (13), and irrelevant study content (159). Subsequently, 135 full-text articles were reviewed, and 126 were excluded due to poor paper quality (12), lack of relevance to sports research (88), or absence of focus on movement speed (26). Ultimately, nine studies met the inclusion criteria for qualitative analysis and were further included in the quantitative synthesis.

### Study quality assessment

The quality of the included studies was assessed using the PEDro scale, with results summarized in Table 3. The average PEDro score for the included studies was 3.67 (range 3–4), indicating a moderate quality level among the selected studies. Although none of the studies fully met all PEDro criteria, all studies satisfied the eligibility criteria, demonstrated baseline group similarity, inter-group comparison, point estimates, and variability. Six of the included studies applied random allocation for participants, yet none met criteria for allocation concealment, blinding of participants, therapists, or assessors, nor intention-to-treat analysis. Given that these studies involved strength training conducted alongside sport-specific skill training, the inherent risks and competition demands made it challenging to ensure participant, therapist, or assessor blinding. Nevertheless, the results showed improvements in movement speed among participants.

**TABLE 3:** Methodological quality assessment scores.

Study	Zhang. et al. (2019)	Zhang. (2016)	Jönhagen et al. (2009)	Fiorilli et al. (2020)	Chaabene et al. (2022)	di Cagno et al. (2020)	Sagelv et al. (2020)	O’Brien et al. (2020)	Stojanovićet al. (2021)	Total
Eligibility criteria	1	1	1	1	1	1	1	1	1	9
Random allocation	1	0	1	1	0	1	1	0	1	6
Allocation concealment	0	0	0	0	0	0	0	0	0	0
Group similar at baseline	1	1	1	1	1	1	1	1	1	9
Blind therapist	0	0	0	0	0	0	0	0	0	0
Blind assessor	0	0	0	0	0	0	0	0	0	0
Follow-Up	0	0	0	0	0	0	0	0	0	0
Intention to treat analysis	0	0	0	0	0	0	0	0	0	0
Between group comparisons	1	1	1	1	1	1	1	1	1	9
Point Measure and Variability	1	1	1	1	1	1	1	1	1	9
PEDro	4	3	4	4	3	4	4	3	4	

The most consistently met criteria were eligibility criteria (n = 9), baseline group similarity (n = 9), inter-group comparison (n = 9), point estimates, and variability (n = 9), as well as random allocation (n = 6). None of the studies satisfied requirements for allocation concealment, participant blinding, therapist blinding, assessor blinding, or intention-to-treat analysis.

### Population characteristics

Table 2 summarizes the characteristics of participants in the nine included studies. Specific details are as follows: (1) Athlete Classification: All nine studies involved athletes, including soccer players (Jönhagen et al., 2009; Fiorilli et al., 2020; Sagelv et al., 2020), basketball players (O’Brien et al., 2020; Stojanović et al., 2021), volleyball players (Zhang, 2016), tennis players (Zhang et al., 2019), fencers (di Cagno et al., 2020), and handball players (Chaabene et al., 2020). (2) Sample Size: Across the studies, there were 253 athletes, with sample sizes ranging from a minimum of 19 (Chaabene et al., 2020) to a maximum of 54 ((di Cagno et al., 2020), averaging 29.8 participants per study. (3) sex: Three studies used female athletes as participants (Zhang, 2016; O’Brien et al., 2020; Chaabene et al., 2020), while the other six studies focused on male athletes (Jönhagen et al., 2009; Fiorilli et al., 2020; Sagelv et al., 2020; Stojanović et al., 2021; Zhang, 2016; Zhang et al., 2019; di Cagno et al., 2020; Chaabene et al., 2020). (4) Age: Three studies involved adolescent athletes (Zhang et al., 2019; Fiorilli et al., 2020; Chaabene et al., 2020), while the remaining six studies focused on adult athletes (O’Brien et al., 2020; Jönhagen et al., 2009; Sagelv et al., 2020; Stojanović et al., 2021; Zhang, 2016; di Cagno et al., 2020). The average participant age across the studies ranged from 13.21 to 23.17 years.

### Characteristics of interventions

The interventions across the nine studies were characterized based on the following aspects: (1) Training Duration: Intervention periods for eccentric training ranged from a minimum of 4 weeks (O’Brien et al., 2020) to a maximum of 8 weeks (Jönhagen et al., 2009; Zhang et al., 2019; Chaabene et al., 2020). (2) Session Duration: Five studies specified session durations, with the shortest being 45 min (Zhang et al., 2019) and the longest being 130 min (Zhang, 2016). (3) Training Frequency: All studies reported training frequency, with a minimum of 1–2 sessions per week (Stojanović et al., 2021) and a maximum of 5–6 sessions per week (Chaabene et al., 2020), while one study adjusted frequency based on participant proficiency (Sagelv et al., 2020).

## Results and measures

The results of this study were grouped based on the impact of eccentric training on athletes’ movement speed. All authors participated in the categorization of the studies. Any disagreements that arose during the categorization process were resolved through discussion and consultation among the authors until a consensus was reached.

### The impact of eccentric training on sport-specific movement speed

Among the nine studies included, three specifically explored the effects of eccentric training on athletes’ sport-specific movement speed (Zhang, 2016; Zhang et al., 2019; di Cagno et al., 2020). The assessment metrics varied, covering tennis-specific movement speed during strokes (Zhang, 2016), volleyball-specific movement abilities (Zhang et al., 2019), and both fencing sprint distance and time over a set distance ((di Cagno et al., 2020). One study confirmed that eccentric training alone improved sport-specific movement speed (Zhang, 2016). Two other studies used eccentric training combined with sport-specific skill training, demonstrating significant effects on movement speed (Zhang et al., 2019; di Cagno et al., 2020). Participants included fencers ((di Cagno et al., 2020), volleyball players (Zhang, 2016), and youth tennis players (Zhang et al., 2019). In two studies, the intervention combined eccentric training with specific sport skills training (Zhang, 2016; Zhang et al., 2019), while one study relied solely on eccentric training ((di Cagno et al., 2020). Two studies conducted a 6-week intervention (Zhang et al., 2019; di Cagno et al., 2020), while one lasted for 8 weeks (Zhang, 2016). Results from these studies suggest that eccentric training, either alone or combined with sport-specific skill training, effectively improves athletes’ sport-specific movement speed.

### The impact of eccentric training on change of direction (COD) ability

Five of the nine studies analyzed examined the effect of eccentric training on change of direction (COD) ability (Zhang et al., 2019; Chaabene et al., 2020; Fiorilli et al., 2020; O'Brien et al., 2022; Stojanović et al., 2021). Three studies used COD-focused metrics as measurement indicators (Chaabene et al., 2020; Fiorilli et al., 2020; O'Brien et al., 2022), while two studies combined sport-specific skill tests as indicators of COD ability. One study incorporated tennis stroke technique with fan-shaped movement as a COD measure (Zhang et al., 2019), and another used a *t*-test run combined with basketball-specific skills (Stojanović et al., 2021). Participants included soccer players (Fiorilli et al., 2020), handball players (Chaabene et al., 2020), and basketball players (O’Brien et al., 2020; Stojanović et al., 2021). Two studies employed a combination of eccentric training with conventional training as an intervention (Chaabene et al., 2020; Fiorilli et al., 2020), while two studies focused solely on eccentric training (O’Brien et al., 2020; Chaabene et al., 2020). Intervention durations varied, with two studies lasting 8 weeks (Chaabene et al., 2020; O’Brien et al., 2020) and one 6 weeks (Fiorilli et al., 2020). Findings from these four studies reveal that eccentric training, particularly when combined with sport-specific skills training, can enhance athletes’ COD ability.

### The impact of eccentric training on sprint speed

Of the nine studies reviewed, seven addressed the impact of eccentric training on short-distance sprint performance (Zhang et al., 2019; Jönhagen et al., 2009; Fiorilli et al., 2020; Sagelv et al., 2020; O’Brien et al., 2020; Stojanović et al., 2021; Chaabene et al., 2020). Two studies used a 5-m sprint as the performance indicator (Stojanović et al., 2021; Chaabene et al., 2020), while four studies used a 10-m sprint (O’Brien et al., 2020; Sagelv et al., 2020; Chaabene et al., 2020; Zhang et al., 2019), including one study incorporating a deceleration phase following the 10-m sprint (Zhang et al., 2019). Two studies measured sprint speed over 20 m (Stojanović et al., 2021; Chaabene et al., 2020), two others over 30 m (Zhang et al., 2019; Jönhagen et al., 2009), and one used a 60-m sprint (Fiorilli et al., 2020). Participants included soccer players (Jönhagen et al., 2009; Fiorilli et al., 2020; Sagelv et al., 2020), basketball players (O’Brien et al., 2020; Stojanović et al., 2021), volleyball players (Zhang et al., 2019), and handball players (Chaabene et al., 2020). Of these studies, five employed eccentric training as the primary intervention (Jönhagen et al., 2009; Sagelv et al., 2020; O’Brien et al., 2020; Stojanović et al., 2021; Zhang et al., 2019), while two used a combination of eccentric and other training modalities (Fiorilli et al., 2020; Chaabene et al., 2020). Intervention lengths ranged from 8 weeks in three studies (Jönhagen et al., 2009; Stojanović et al., 2021; Chaabene et al., 2020) to 4 weeks in one study (O’Brien et al., 2020), with others lasting 6 weeks (Fiorilli et al., 2020; Sagelv et al., 2020). Results indicate that eccentric training significantly improves athletes’ 5-m sprint speed and has notable effects on 10-m sprint speed. For 20-m and 30-m sprint speeds, significant improvements were observed in only one study each (Chaabene et al., 2020; Jönhagen et al., 2009), while 60-m sprint speed also showed marked improvement in one study (Fiorilli et al., 2020).

## Discussion

This systematic review aimed to investigate the effects of eccentric training on athletes’ movement speed. Based on the screening criteria, nine studies were included in the final analysis. These studies indicate that eccentric training can improve athletes’ sport-specific movement speed and change of direction (COD) ability to some extent. Furthermore, eccentric training positively impacts sprint speed, particularly over 5 and 10 m, with moderate effects on 20- and 30-m sprints. This finding aligns with previous reviews (Liu et al., 2020), which support the role of eccentric training in enhancing movement speed. Detailed analysis follows the “Results and Measures” framework.

### Eccentric Training’s impact on sport-specific movement speed

In competitive sports, sport-specific speed is crucial for achieving high-level performance (Douglas et al., 2017). This review includes three studies analyzing the effects of eccentric training on sport-specific movement speed. Participants across the studies included fencers ((di Cagno et al., 2020), volleyball players (Zhang, 2016), and youth tennis players (Zhang et al., 2019). Analysis of experimental and control groups in these studies shows a statistically significant improvement in sport-specific movement speed, with p-values ranging from 0.02 to 0.000. The results align with prior findings, such as those by Guex and Millet (2013), who found that high-load eccentric training improved sport-specific speed and injury prevention in athletes. Similarly, Gonzalo-Skok et al. (2017) reported significant improvements in dribbling speed for amateur soccer players following high-load eccentric training.

In these studies, eccentric training improves athletes’ movement by enhancing muscle length, tension coordination, joint stability, and stretch-shortening cycle efficiency. This adaptation improves athletes’ posture control, deceleration abilities, and speed control, benefitting rapid directional changes and stops (Zhang et al., 2019; di Cagno et al., 2020; Zhang, 2016). However, none of these studies addressed eccentric training’s impact on upper limb-specific movement speed. Upper limb movement speed is also vital in competitive sports, as in volleyball arm swings or golf club swings. Future research could explore eccentric training’s application in upper-limb training to expand its use.

### Eccentric Training’s impact on change of direction (COD) ability

In competitive sports, COD ability influences performance and reduces injury risk (Bishop et al., 2021; Mijatovic et al., 2022). This review includes five studies on the effects of eccentric training on athletes’ COD abilities. The participants were soccer players (Fiorilli et al., 2020), handball players (Chaabene et al., 2020), basketball players (O'Brien et al., 2022; Stojanović et al., 2021), and volleyball players (Zhang et al., 2019). Among these studies, three reported significant improvements in COD ability (p-values ranging from 0.000 to 0.020; Zhang et al., 2019; Fiorilli et al., 2020; Stojanović et al., 2021), while effect sizes (ES) ranged from 0.32 to 1.38 (Chaabene et al., 2020; O'Brien et al., 2020), indicating a substantial positive effect of eccentric training on COD speed. This finding is supported by Núñez et al. (2018), who found that unilateral and bilateral eccentric overload training significantly enhanced COD ability in healthy males. A systematic review on eccentric overload training also concluded that it positively affects COD (Liu et al., 2020). Eccentric training improves eccentric strength in the knee and hip joints, reduces athletes’ inertia, and shortens deceleration time, aiding lateral movements and cuts. These results are consistent with findings by Chaabene et al. (2018), who reported mechanical and kinematic adaptations during COD tasks. The studies reviewed utilized randomized trials to confirm eccentric training’s impact on COD, adding value to future research. Additionally, one study combined eccentric training with functional training (Zhang et al., 2019), broadening its application. However, limitations include small sample sizes and limited participant diversity, affecting generalizability. Future studies should recruit larger, more representative samples and explore eccentric training’s effects across various angles and distances. In-depth analysis from biomechanical and biological perspectives would enhance understanding of how eccentric training influences COD ability.

### Eccentric Training’s impact on sprint speed

Speed is a critical factor in athlete selection and evaluation (Vrublevskiy et al., 2020). Among the nine studies reviewed, seven assessed the impact of eccentric training on sprint speed. Significant improvements were observed in 5-m sprint speed (p = 0.01; Stojanović et al., 2021; ES = 1.07; Chaabene et al., 2020), and 10-m sprint speed (p = 0.003; O’Brien et al., 2020; ES = 0.54–0.66; Chaabene et al., 2020; Sagelv et al., 2020). Moderate effects were observed for 20-m sprints (ES = 0.53; Chaabene et al., 2020), while others showed no significant effect (p = 0.88; Stojanović et al., 2021). Similar partial effects were found in 30-m sprint speed (p = 0.000; Jönhagen et al., 2009, but p = 0.46 for Zhang et al., 2019), while a significant effect was found in 60-m sprint speed (p = 0.001; Fiorilli et al., 2020). Participants included tennis (Zhang et al., 2019), soccer (Fiorilli et al., 2020; Sagelv et al., 2020), handball (Chaabene et al., 2020), and basketball players (O’Brien et al., 2020; Stojanović et al., 2021). The findings align with Nevado et al. (2021), who showed that eccentric overload training and small-sided games significantly impacted acceleration and deceleration performance in female soccer players. Cook, Beaven & Kilduff (2013) found similar improvements in maximum speed after 3 weeks of eccentric and overspeed training in amateur athletes. Eccentric training improves athletes’ acceleration by enhancing muscle strength and explosiveness (Fiorilli et al., 2020). Additionally, studies show that eccentric training impacts neuromuscular control (O’Brien et al., 2020; Stojanović et al., 2021). However, not all studies found significant impacts on 20- and 30-m speeds, indicating that eccentric training may not benefit all sports equally. Future studies should include diverse samples to explore the differential effects of eccentric training across various sports and distances. Additionally, analyzing the mechanisms behind eccentric training’s impact on sprint speed from physiological and biomechanical perspectives could deepen our understanding.

## Limitations of the study

This systematic review has several limitations that should be considered. Firstly, although this review includes a considerable number of studies on various sports, all studies analyzed the effects of eccentric training on lower-limb movement speed, with no studies addressing the impact on upper-limb speed. Secondly, only one study specifically focused on female athletes, which limits the understanding of eccentric training’s effects on movement speed enhancement across sexes. Thirdly, only two studies used eccentric training as the sole intervention, while the other seven combined it with other training methods, potentially confounding the effects of eccentric training with those of additional interventions.

## Conclusion

The analysis of the included studies suggests that eccentric training significantly improves athletes’ sport-specific movement (in fencing, volleyball, and tennis), change of direction (COD) ability, and short-distance sprint speed. Further studies are necessary to fully evaluate the mechanisms through which eccentric training impacts movement speed and to include a broader range of sports, thereby extending the application scope of eccentric training.

## Data Availability

The raw data supporting the conclusions of this article will be made available by the authors, without undue reservation.
